# Genome sequence of the anaerobic bacterium *Bacillus* sp. strain ZYK, a selenite and nitrate reducer from paddy soil

**DOI:** 10.4056/sigs.3817480

**Published:** 2014-03-15

**Authors:** Peng Bao, Jian-Qiang Su, Zheng-Yi Hu, Max M. Häggblom, Yong-Guan Zhu

**Affiliations:** 1 State Key Lab of Urban and Regional Ecology, Research Center for Eco-Environmental Sciences, Chinese Academy of Sciences, Beijing, P. R. China; 2 Key Lab of Urban Environment and Health, Institute of Urban Environment, Chinese Academy of Sciences, Xiamen, P. R. China; 3 College of Resources and Environment, University of Chinese Academy of Sciences, Beijing, China; 4 Rutgers University, Department of Biochemistry and Microbiology, School of Environmental and Biological Sciences, New Brunswick, NJ, USA

**Keywords:** anaerobic, spore-forming, Gram-positive, nitrate-reduction, selenite-reduction, arsenic resistance, paddy soil, *Bacillaceae*

## Abstract

*Bacillus* sp. strain ZYK, a member of the phylum *Firmicutes*, is of interest for its ability to reduce nitrate and selenite and for its resistance to arsenic under anaerobic conditions. Here we describe some key features of this organism, together with the complete genome sequence and annotation. The 3,575,797 bp long chromosome with its 3,454 protein-coding and 70 RNA genes, and the information gained from its sequence will be relevant to the elucidation of microbially-mediated transformations of nitrogen, selenium and arsenic in paddy soil.

## Introduction

*Bacillus* sp. ZYK (=DSM 26460 =CGMCC 1.5179) was isolated from a paddy soil in Dehong, Yunnan, China and is an anaerobic nitrate-reducing, Gram-positive bacterium [1]. Strain ZYK belongs to the genus *Bacillus*, and based on 16S rRNA phylogeny, is most closely related to *Bacillus azotoformans* isolated from garden soil, which is capable of reducing nitrate, nitrite, nitrous oxide, and nitric oxide under anaerobic conditions [2-4]. Strain ZYK is capable of nitrate-reduction under anaerobic conditions and, in addition, demonstrated selenite-reducing ability and arsenic resistance (unpublished data). *Bacillus* spp. are commonly found in paddy soil and may play important roles in elemental cycling during periodically changing redox conditions [5-8]. Therefore, strain ZYK is a suitable model for studying the properties of genes involved in denitrification, selenite-reduction and arsenic resistance pathways of paddy soil bacteria. Here we summarize the features of *Bacillus* sp. strain ZYK and provide a description of its sequenced genome, now available for detailed analysis.

## Classification and features

**Figure 1:**
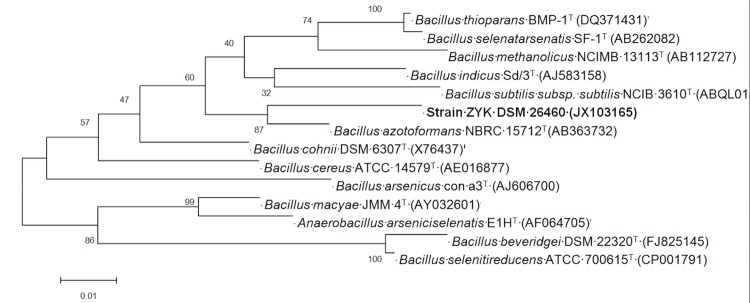
Phylogenetic tree highlighting the position of *Bacillus* sp. ZYK relative to selected *Bacillus* species. The strains and their corresponding GenBank accession numbers of 16S rRNA genes are as indicated. The tree, based on 1,545 positions, was built with MEGA 5 [11] using the Maximum likelihood method. Bar: 0.01substitutions per nucleotide position.

Based on 16S rRNA gene phylogeny and genome information, strain ZYK was a member of the genus *Bacillus*, most closely related to *Bacillus azotoformans* (AB363732), with a sequence similarity of 96.3% based on a Blast analysis [9] of the most recent release of the Greengenes database [10]. A phylogenetic tree (Figure 1) was constructed using the Maximum likelihood method under the default settings for complete sequences of genes encoding 16S rRNA derived from sequenced genomes of *Bacillus* spp., along with the sequences of representative members of the genus.

**Figure 2:**
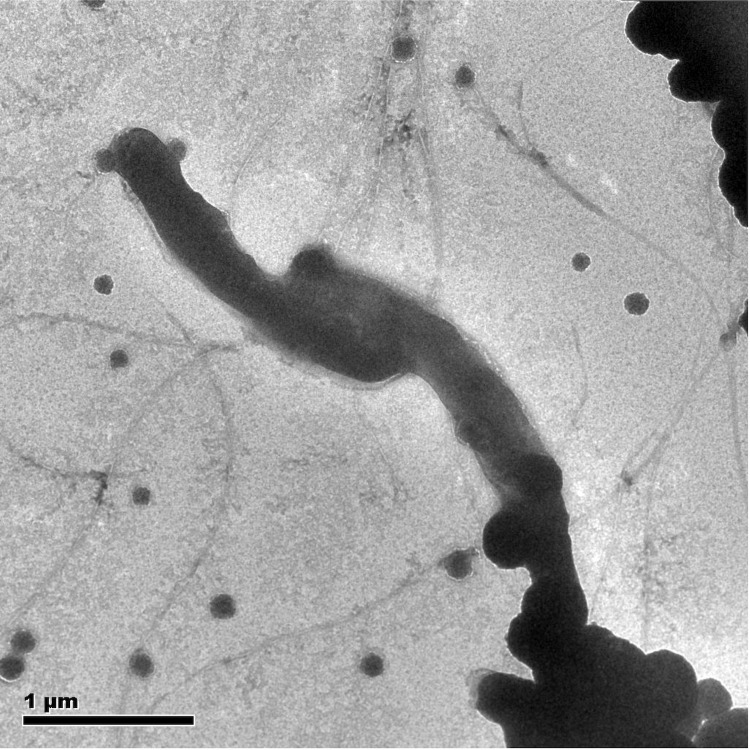
Transmission electron microscopy of strain ZYK. Scale bar corresponds to 1.0 μm.

Strain ZYK is an anaerobic, Gram-positive, spore-forming, motile, rod-shaped (0.2-0.3 µm wide and 1.5-2.0 µm long) (Figure 2). The strain grew optimally at pH 7.0-7.2 (range 6.0-7.6), 30-40°C (range 21-45°C) and at low salinity (NaCl range 0-1.1%) (Table 1) in freshwater anaerobic medium [24]. On anaerobic LB agar, strain ZYK forms small, white colonies with entire edges (data not shown). Carbon substrates utilized for growth by strain ZYK included D-glucose, maltose, lactose, and sucrose. Strain ZYK reduces nitrate and selenite under anaerobic conditions in freshwater medium.

**Table 1:** Classification and general features of strain ZYK according to the MIGS recommendations [1]

**MIGS ID**	**Property**	**Term**	**Evidence codes**
	Classification	Domain *Bacteria*	TAS [12]
		Phylum *Firmicutes*	TAS [13-15]
		Class *Bacilli*	TAS [16,17]
		Order *Bacillales*	TAS [18,19]
		Family *Bacillaceae*	TAS [18,20]
		Genus *Bacillus*	TAS [18,21,22]
		Strain ZYK	IDA
	Gram stain	Positive	IDA
MIGS-37.1	Cell shape	Rod-shaped	NAS
MIGS-37.2	Motility	Motile	NAS
MIGS-37.3	Sporulation	Sporulating	NAS
MIGS-37.9	Cell arrangement	Single	NAS
MIGS-37.12	Optimum pH	7.0	NAS
MIGS-6	Optimum temperature	30°C	NAS
	Salinity	0-1.1%	IDA
MIGS-22	Oxygen requirement	Strict	NAS
	Carbon source	D-Glucose, Maltose, lactose, sucrose	IDA
MIGS-6	Habitat	Paddy soil	NAS
MIGS-15	Biotic relationship	Free-living	NAS
	Pathogenicity	None-pathogen	NAS
	Biosafety level	1	NAS
MIGS-4	Geographic location	Dehong, Yunnan, China	NAS
MIGS-4.1	Latitude	24^o^64´70"N	NAS
MIGS-4.2	Longitude	98^o^53´45"E	NAS
MIGS-4.5	Isolation	Paddy soil	NAS

Evidence codes - IDA: Inferred from Direct Assay; TAS: Traceable Author Statement (i.e., a direct report exists in the literature); NAS: Non-traceable Author Statement (i.e., not directly observed for the living, isolated sample, but based on a generally accepted property for the species, or anecdotal evidence). These evidence codes are from the Gene Ontology project [23]. If the evidence code is IDA, the property was directly observed by one of the authors.

### Genome project history

*Bacillus* sp. ZYK was selected for sequencing because of its phylogenetic affiliation with a lineage of paddy soil bacteria that may influence elemental cycling in paddy fields. The genome project is deposited in the Genomes OnLine Database (GOLD) as project Gi22906, and the complete genome sequence is in GenBank under accession number ANOK00000000 (Table 2). A summary of the main project information is shown in Table 2.

**Table 2:** Genome sequencing project information

**MIGS ID**	**Property**	**Term**
MIGS-31	Finishing quality	Complete
MIGS-28	Libraries used	Two libraries 500 bp PCR-free library, 2000 bp index library
MIGS-29	Sequencing platforms	Illumina
MIGS-31.2	Fold coverage	140×
MIGS-30	Assemblers	SOAPdenovo 1.05
MIGS-32	Gene calling method	Glimmer 3.0
	Locus TAG	D612
	Genbank ID	ANOK00000000
	Genbank Date of Release	January 15, 2013
	GOLD ID	Gi22906
	NCBI taxon ID	1191699
MIGS-13	Source material identifier	DSMZ 26460, CGMCC 1.5179
MIGS-38.2	Project relevance	Agricultural, Bioremediation, Environmental

### Growth conditions and DNA isolation

For the preparation of genomic DNA, one colony was picked from an anaerobic LB agar plate, and grown in anaerobic freshwater medium at 30°C [24]. A culture (1.0 ml) at 0.6 OD_600nm_ was inoculated into 100 ml of anaerobic freshwater media. Cells were collected by centrifugation after growing to 0.6 OD_600nm_. Cells were suspended in TE buffer (10 mM NaCl, 20 mM Tris-HCl, 1.0 mM EDTA, pH 8.0), and treated with lysozyme to lyse the cell wall. SDS and proteinaseK were added to denature and degrade proteins. Cell lysates were extracted with phenol-chloroform and the nucleic acids were precipitated by addition of isoamylol. The nucleic acid pellet was washed with 100% ethanol, dissolved in double distilled water and then treated with RNase A [25].

### Genome sequencing and assembly

The genome of ZYK was sequenced at the Beijing Genomics Institute (BGI) using Illumina paired-end sequencing. Draft assemblies were based on 4,233,334 reads totaling 380 Mb of 500 bp PCR-free library and 2,184,080 reads totaling 196 Mb of 2,000 bp index library. The SOAPdenovo software package independently developed by BGI (version 1.05 [26],) was used for sequence assembly and quality assessment. To achieve optimal assembly results, the key parameter K was set at 43 after several adjustments. Gaps between contigs were closed by KRSKGF software, version 1.2 (independently developed by BGI) and Gapcloser, version 1.10. The complete nucleotide sequence of *Bacillus* sp. strain ZYK and its annotation can be found online at the IMG (Integrated Microbial Genome) portal of JGI [27], as well at the genome resource site of NCBI [28].

### Genome annotation

Genes were identified using Glimmer, version 3.0 [29]. The predicted CDSs were translated and used to search KEGG, COG, SwissPort, TrEMBL, NR and GO databases. These data sources were combined to assert a product description for each predicted protein. Transposons were identified using RepeatMaster (with Repbase) and RepeatProteinMasker (with its own database) software. Tandem repeat sequences were predicted by TRF (Tandem Repeat Finder) software. The rRNA, tRNA and sRNA were predicted by using rRNAmmer [30], tRNAscan [31] and Rfam [32] software, respectively.

## Genome properties

**Figure 3:**
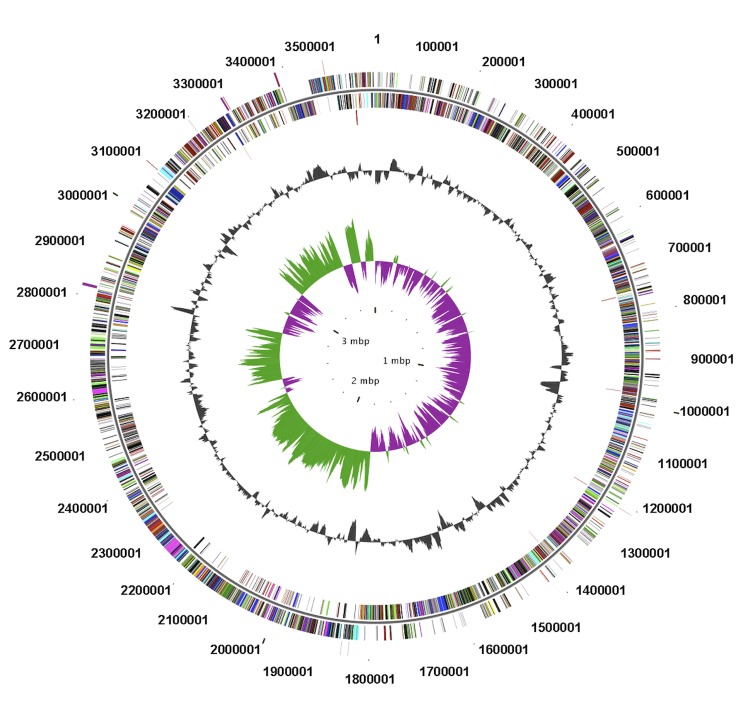
Graphical representation of circular map of the chromosome of strain ZYK. From outside to the center: Genes on forward strand (colored by COG categories), Genes on reverse strand (colored by COG categories), RNA genes (tRNAs green, rRNAs red, other RNAs black), GC content, GC skew.

The genome consists of a circular chromosome of 3,575,797 bp in size with a GC content of 36.1% (Figure 3, Table 3). Of the 3,454 predicted genes, 70 are RNA genes, 136 are secreted protein coding genes, and 3,318 are non-secreted protein coding genes. Of the total predicted genes, 2,030 represent COG functional categories. The distribution of genes into COG functional categories is presented in Figure 3 and Table 4.

**Table 3:** Genome statistics

**Attribute**	**Value**	**% of total**
Genome size (bp)	3,575,797	100.00
DNA coding region (bp)	3,002,982	83.98
DNA G+C content (bp)	1,290,862	36.10
Total genes	3454	100.00
RNA genes	70	2.03
Protein-coding genes (bp)	3,002,982	83.98
Genes with function prediction	3261	94.41
Genes assigned to COGs	2,030	58.77
Genes assigned to Pfam domains (bp)	617,696	17.27
Genes with signal peptides	169	4.89
Genes with transmembrane helices	132	3.82
CRISPR repeats	84	0.09

**Table 4:** Number of genes associated with the 25 general COG functional categories

**Code**	**Value**	**%age^a^**	**Description**
J	149.0	6.5	Translation
A	0.0	0.0	RNA processing and modification
K	164.0	7.1	Transcription
L	119.0	5.2	Replication, recombination and repair
B	1.0	0.04	Chromatin structure and dynamics
D	27.0	1.2	Cell cycle control, mitosis and meiosis
Y	0.0	0.0	Nuclear structure
V	24.0	1.0	Defense mechanisms
T	162.0	7.0	Signal transduction mechanisms
M	95.0	4.1	Cell wall/membrane biogenesis
N	75.0	3.3	Cell motility
Z	0.0	0.0	Cytoskeleton
W	0.0	0.0	Extracellular structures
U	44.0	1.9	Intracellular trafficking and secretion
O	97.0	4.2	Posttranslational modification, protein turnover, chaperones
C	155	6.7	Energy production and conversion
G	79.0	3.4	Carbohydrate transport and metabolism
E	239.0	10.4	Amino acid transport and metabolism
F	61.0	2.7	Nucleotide transport and metabolism
H	93.0	4.0	Coenzyme transport and metabolism
I	97.0	4.2	Lipid transport and metabolism
P	127.0	5.5	Inorganic ion transport and metabolism
Q	38.0	1.7	Secondary metabolites biosynthesis, transport and catabolism
R	261.0	11.3	General function prediction only
S	193.0	8.4	Function unknown
-	1424	41.2	Not in COGs

a) The total is based on the total number of protein coding genes in the annotated genome.

## Insights into the genome sequence

*Bacillus* sp. ZYK can reduce nitrate and selenite under anaerobic conditions (unpublished data). The inspection of the genome of strain ZYK confirmed the presence of nitrate reductase coding genes, in support of the physiological data. Genes for a respiratory nitrate reductase corresponding to a heterotrimeric structure with four subunits, including *narG*, *narH*, *narI* and *narJ* present in the genome of strain ZYK. Genes encoding a second type of nitrate reductase, Nap (periplasmic nitrate reductase) including *napA*, *napB*, and *napD* were also found in the ZYK genome. We also identified in the genome a formate-dependent nitrite reductase coding gene (*nrfA*) and a copper-containing nitrite reductase coding gene (*nirK*).

An arsenate reductase coding gene (*arsC*) was identified with 77% similarity to the *Bacillus megaterium arsC* gene (AJ515540). An arsenite efflux pump gene was also identified as *arsB* with 78% similarity to *Bacillus* sp. CDB3 *arsB* gene (AF178758.2). Two DMSO reductase genes have 59.2% and 60.3% similarity with *Desulfosporosinus orientis* DMSO reductase (Fe-S cluster containing hydrogenase coding gene) and *Bacillus* sp. 1NLA3E DMSO reductase (dimethylsulfoxide reductase, chain B), respectively. The discovery of an arsenate reductase coding gene (*arsC*) and DMSO reductase sequences suggests that the reduction capabilities of strain ZYK are broader than expected, and that other substrates be tested. Particularly, we are interested in determining whether selenite reduction activity in ZYK is mediated by a hydrogenase [33], a nitrite reductase [34] or a DMSO reductase. While the reduction of selenite to elemental selenium is a common feature of diverse microorganisms, the genes responsible for this process remain largely uncharacterized and virtually nothing is known about their regulation [33-35], or their interactions with other respiratory pathways. In addition to *Bacillus sp.* ZYK, the genomes of two bacteria capable of selenite reduction, *Bacillus selenitireducens* (NC_014219.1) [36] and *Desulfirispirillum indicum* S5 [37,38], have been sequenced. The investigation of the functional genes of strain ZYK will consequently enhance the understanding of the electron acceptor utilization pathways in microorganisms, and how nitrogen, selenium and arsenic cycling is mediated by microorganisms active in paddy soil. Further study of these reductase gene-coding sequences may reveal the importance of the *Bacillus* genus in elemental cycling in paddy soils.
